# COVID-19 Detection Using Deep Learning Algorithm on Chest X-ray Images

**DOI:** 10.3390/biology10111174

**Published:** 2021-11-13

**Authors:** Shamima Akter, F. M. Javed Mehedi Shamrat, Sovon Chakraborty, Asif Karim, Sami Azam

**Affiliations:** 1 Department of Bioinformatics and Computational Biology, George Mason University, Fairfax, VA 22030, USA; sakter5@gmu.edu; 2 Department of Software Engineering, Daffodil International University, Dhaka 1207, Bangladesh; javedmehedicom@gmail.com; 3 Department of Computer Science and Engineering, Ahsanullah University of Science and Technology, Dhaka 1208, Bangladesh; sovonchakraborty2014@gmail.com; 4 College of Engineering, IT and Environment, Charles Darwin University, Casuarina, NT 0909, Australia; asif.karim@cdu.edu.au

**Keywords:** COVID-19, chest X-ray image, CNN, Mobilenetv2, modified MobileNetV2, performance evaluation

## Abstract

**Simple Summary:**

The study proposes an automated deep learning-based classification model, based on a Convolutional Neural Network, that demonstrates a rapid detection rate for COVID-19. The training dataset consists of 3616 COVID-19 chest X-ray images and 10,192 healthy chest X-ray images which were then augmented. Initially using the dataset, the symptoms of COVID-19 were detected by employing eleven existing CNN models. MobileNetV2 showed enough promise to make it a candidate for further modification. The resulting model produced the highest accuracy of 98% in classifying COVID-19 and healthy chest X-rays among all the implemented CNN models. The results suggest that the proposed method can efficiently identify the symptoms of infection from chest X-ray images better than existing methods.

**Abstract:**

COVID-19, regarded as the deadliest virus of the 21st century, has claimed the lives of millions of people around the globe in less than two years. Since the virus initially affects the lungs of patients, X-ray imaging of the chest is helpful for effective diagnosis. Any method for automatic, reliable, and accurate screening of COVID-19 infection would be beneficial for rapid detection and reducing medical or healthcare professional exposure to the virus. In the past, Convolutional Neural Networks (CNNs) proved to be quite successful in the classification of medical images. In this study, an automatic deep learning classification method for detecting COVID-19 from chest X-ray images is suggested using a CNN. A dataset consisting of 3616 COVID-19 chest X-ray images and 10,192 healthy chest X-ray images was used. The original data were then augmented to increase the data sample to 26,000 COVID-19 and 26,000 healthy X-ray images. The dataset was enhanced using histogram equalization, spectrum, grays, cyan and normalized with NCLAHE before being applied to CNN models. Initially using the dataset, the symptoms of COVID-19 were detected by employing eleven existing CNN models; VGG16, VGG19, MobileNetV2, InceptionV3, NFNet, ResNet50, ResNet101, DenseNet, EfficientNetB7, AlexNet, and GoogLeNet. From the models, MobileNetV2 was selected for further modification to obtain a higher accuracy of COVID-19 detection. Performance evaluation of the models was demonstrated using a confusion matrix. It was observed that the modified MobileNetV2 model proposed in the study gave the highest accuracy of 98% in classifying COVID-19 and healthy chest X-rays among all the implemented CNN models. The second-best performance was achieved from the pre-trained MobileNetV2 with an accuracy of 97%, followed by VGG19 and ResNet101 with 95% accuracy for both the models. The study compares the compilation time of the models. The proposed model required the least compilation time with 2 h, 50 min and 21 s. Finally, the Wilcoxon signed-rank test was performed to test the statistical significance. The results suggest that the proposed method can efficiently identify the symptoms of infection from chest X-ray images better than existing methods.

## 1. Introduction

The COVID-19 outbreak has risen to the status of one of the most severe public health issues of the last several years. The virus spreads rapidly: the reproduction number of COVID-19 varied from 2.24 to 3.58 during the initial months of the pandemic [1], indicating that each infected individual on average transmitted the disease to two or more others. Consequently, the number of COVID-19 infections grew up from a few hundred cases (most of them in China) in January 2020 to more than 43 million cases in November 2020 disseminated across the world [2].

It’s believed that the coronavirus that causes COVID-19 is the same one that causes SARS-COV2 and MERS (MERS) [3]. COVID-19 has a wide range of symptoms that appear after an average incubation period of 5.2 days. Fever, dry cough, and tiredness are common symptoms, while others include headache, hemoptysis, diarrhea, dyspnea, and lymphopenia [4,5]. In December 2019, the first human was infected with coronavirus (SARS-COV-2), and it is mostly transmitted by droplets produced when infected people talk, cough, or sneeze [6,7,8,9,10,11]. Because the droplets are too heavy to travel far, they can only be propagated via direct contact [12]. Recent research estimates that the COVID-19 can survive up to 3 h in the air, 4 h on copper, and 72 h on plastic and stainless steel, but the precise durations are yet unknown. However, the general health research community is yet to agree on answers to these issues, which are still under study. An infection with COVID-19 affects the tissues of the lungs. Some affected people may not notice any symptoms in the early stages, while fever and cough were the most common core symptoms for the majority of patients. Other side effects include muscle pains, a sore throat, and a headache. Cough medicine, pain killers, fever reducers, and antibiotics are provided to patients based on their symptoms, not the disease organism. The patient must be hospitalized and treated in an Intensive Care Unit (ICU), which may include the use of a ventilator to help the patient breathe [13]. As a result of its severity and ease of transmission, COVID-19 has spread rapidly across the world. The greater effect on health care departments is primarily due to the number of individuals impacted day by day, as they need to give mechanical ventilators to critically ill patients admitted to ICU. As a result, the number of ICU beds must be significantly expanded. In the aforementioned scenario, early diagnosis is critical in ensuring patients receive appropriate treatment, while reducing the load on the healthcare system. COVID-19 is still a deadly disease due to the absence of early diagnostic techniques around the world, as well as having medical preconditions such as cancer, chronic liver, lung, and kidney diseases, and diabetes. Though RT-PCR diagnosis techniques are available in most parts of the world, under-developed countries still cannot afford to test all their people promptly [14]. In 2020–2021, this disease claimed the lives of millions of people across the earth. Vaccines for COVID-19 are now being developed in a number of countries. Vaccines produced by Pfizer, AstraZeneca, Moderna, Serum Institute of India Pvt. Ltd., Janssen, Sinopharm, Sinovac are among the vaccines that have been approved by WHO for administration [15]. Such approved vaccines have substantially reduced the deadliness of the disease.

Artificial intelligence has shown its efficiency and excellent performance in automated image categorization issues via various machine learning methods and is currently being used to automate the diagnosis of various diseases [16]. Furthermore, machine learning refers to models that can learn and make decisions based on vast quantities of data samples. Artificial intelligence accomplishes activities that need human intellect, such as voice recognition, translation, visual perception, and more, by performing calculations and predictions depending on the incoming data [17,18]. Deep learning is a collection of machine learning techniques that primarily concentrate on the automated extraction and categorization of image features, and has shown tremendous promise in a variety of applications, particularly in health care [19]. Scientists from around the world are trying to develop technologies that can assist radiologists/doctors in their diagnoses. To identify the optimal network for the area of radiology and medical image processing, a variety of AI methods have been used so far [20,21]. Convolutional Neural Networks (CNNs) and Recurrent Neural Networks (RNNs) are two prominent deep-learning-based networks that have been extensively utilized in medical research fields like speech recognition, computer vision, and Natural Language Processing (NLP), and have often produced commendable results. The classification, localization, and segmentation of images using CNN have shown impressive results in medical image processing [22,23,24,25].

This is a significant step forward in the identification of COVID-19 and other forms of lung inflammation due to artificial intelligence (AI). The WHO recommends using RT-PCR as the main diagnostic method for COVID-19 detection. Chest X-rays or chest CT scans are also widely used, but should only be preferred when the RT-PCR test is not available in a timely manner [14]. In most cases, it is seen that the nasal swap test results vary if done at different times of the day. For instance, the possibility of getting a positive result is higher if the test is done in the morning compared to a test done in the evening [26]. Furthermore, the detection of COVID-19 positive patients was significantly delayed due to a high number of false-negative results [27]. The RT-PCR has a low success rate of 70% [28] and a sensitivity of 60–70% [29,30]. Moreover, the false-negative cases of RT-PCR show positive results on chest X-ray imaging [31]. Due to its widespread availability, X-ray imaging has played a significant role in many medical and epidemiological situations [32,33]. Because of its operating speed, low cost, and ease of use for radiologists, chest X-ray seems to be promising for emergency situations and therapy. A prior study, however, found significant discrepancies in chest X-ray images obtained from patients who had COVID-19 [34].

The study proposed an intelligent deep learning architecture such as a modified MobileNetV2 with RMSprop optimizer to detect COVID-19 disease. For the study, 13,808 X-ray image data were collected from the dataset and augmented to a larger dataset of 52,000 chest X-ray images. Image processing methods such as enhancement, normalization, and data augmentation were used to help the proposed model not only to avoid overfitting but also to demonstrate the highest accuracy. The performance accuracy and compilation time of the newly proposed methods are compared to those of eleven existing CNN models. Finally, to access the statistical significance, a Wilcoxon signed-rank test was performed.

## 2. Literature Review

Artificial intelligence approaches have repeatedly given accurate and dependable outcomes in applications that use image-based data. Using deep learning techniques, researchers have been investigating and analyzing chest X-ray images to identify COVID-19 in recent years.

In [35], the images were normalized to extract enhanced features, which were then fed into image classification algorithms utilizing deep learning techniques. Five cutting-edge CNN systems, VGG19, MobileNetV2, Inception, Xception, and InceptionResNetV2, on a transfer-learning scenario, were tested to detect COVID-19 from control and pneumonia images. Experiments were conducted in two parts: one with 224 COVID-19 pictures, 700 bacterial pneumonia images, and 504 control images, and another with the prior normal and COVID-19 data but 714 instances of bacterial and viral pneumonia. In the two- and three-class classifications, the MobileNetV2 net had the greatest results, with 96.78% and 94.72% accuracy, respectively.

In [36], both VGG16 CNN and Resnet50, which were trained on color camera images from ImageNet, were utilized to perform transfer learning. To assess the feasibility of utilizing chest X-rays to diagnose COVID-19, 10-fold cross-validation was performed to obtain an overall accuracy of 89.2%.

Three CNN architectures (ResNet50, InceptionV3, and InceptionRes-NetV2) were evaluated in relation to COVID-19 identification in [37], utilizing a database of just 50 controls and 50 COVID-19 cases. ResNet50 achieved the highest accuracy of 98%.

In [38], a successful performance in diagnosis accuracy found in this research demonstrates that deep CNNs could correctly and efficiently distinguish 21,152 normal and abnormal chest radiographs. The CNN model pre-trained on datasets of adult patients and fine-tuned on pediatric patients obtained an accuracy of 94.64%, a sensitivity of 96.5% and a specificity of 92.86% for normal versus pneumonia categorization.

For transfer learning in [39], four CNN networks (ResNet18, ResNet50, SqueezeNet, and DenseNet-121) were employed. Experiments were carried out using a database of 184 COVID-19 pictures as well as 5000 no findings and pneumonia images. According to the reported findings, the sensitivity was about 98% and the specificity was 92.9%.

The proposed model in [40] was based on an X-ray image dataset and shows that COVID-CAPS outperforms prior CNN-based models. COVID-CAPS attained an accuracy of 95.7%, a sensitivity of 90%, and a specificity of 95.8% despite having a much lower number of trainable parameters than other models.

In [41], from 400 chest X-ray images, individuals with COVID-19 symptoms were identified using eight different deep learning techniques: VGG16, InceptionResNetV2, ResNet50, DenseNet201, VGG19, MobilenetV2, NasNetMobile, and ResNet15V2. NasNetMobile beat all other models in chest X-ray datasets, attaining an accuracy of 93.94%.

Seven different deep CNN models on a corpus of 50 controls and 25 COVID-19 patients were evaluated by the authors in [42]. The VGG19 and DenseNet models produced the greatest results, with F1 scores of 0.89 and 0.91 for controls and patients, respectively.

The authors of [43] utilized a database of 127 COVID-19, 500 controls, and 500 pneumonia patients collected from various sources for the binary classification of COVID-19 and controls, as well as the multiclass classification of COVID-19, controls, and pneumonia. The Darknet model was modified for transfer-learning and five-fold cross-validation, yielding 98% accuracy in binary classification and 87% accuracy in multiclass classification.

Although [44] presented an Xception transfer learning technique, it was restricted to two multi-class classification tasks: (i) controls vs. COVID-19 vs. viral and bacterial pneumonia; and (ii) controls vs. COVID-19 vs. pneumonia. To resolve the corpus imbalance, an under-sampling technique was used to randomly remove registers from larger classes, resulting in 290 COVID-19, 310 control, 330 bacterial pneumonia, and 327 viral pneumonia chest X-ray images. The stated accuracy was 89% in the four-class scenario and 94% in the three-class condition. Additionally, the accuracy was 90% in a three-class cross-database experiment.

The suggested method in [45] employs a two-phase classification approach based on 2088 (696 normal, 696 pneumonia, and 696 nCOVID-19) and 258 (86 pictures of each category) chest Xray images, respectively. The validation test results obtained were as follows: ACC = 91.329% and AUC = 0.831%.

Paper [46] utilized publicly accessible X-ray images (1583 normal, 4292 pneumonia, and 225 verified COVID-19) to train deep learning (ConvNet) and machine learning classifiers (SVM, LR, NB, DT, and KNN). Thirty-eight experiments used convolutional neural networks (ConvNet), ten experiments used five machine learning models (SVM, LR, NB, DT, and CNN), and fourteen experiments used state-of-the-art pre-trained networks (VGG16, ResNet50, MobileNetV2, and DenseNet121) for transfer learning with eightfold cross-validation. The average sensitivity was 93.84%, the average specificity was 99.18%, and the average accuracy was 98.50%.

The dataset analyzed in [47] includes 1428 chest radiographs of patients with confirmed COVID-19 positivity, common bacterial pneumonia, and healthy individuals (no infection). In this research, the network was successfully trained on relatively tiny chest radiographs using the pre-trained VGG16 model for classification tasks. The trial demonstrated a 96% and 92.5% accuracy rate for two classes (COVID and non-COVID) and three output classes (COVID, non-COVID pneumonia, and normal), respectively.

The article [48] details the procedure for training a Convolutional Neural Network, Grad-CAM, using a dataset of over 79,500 X-ray pictures (controls, pneumonia, or COVID-19 groups) collected from various sources, including over 8500 COVID-19 examples. Three distinct experiments are conducted using three distinct preprocessing methods in order to assess and compare the generated models. The used technique achieves a classification accuracy of 91.5% but needs preprocessing of the pictures for lung segmentation.

In [49], a benchmark X-ray data collection, dubbed QaTa-Cov19, was produced. It contained about 6200 X-ray pictures. The data set contained 462 X-ray pictures of individuals diagnosed with COVID-19, as well as three other classifications: bacterial pneumonia, viral pneumonia, and normal. The suggested CSEN-based classification method, which incorporates feature extraction from a state-of-the-art deep neural network solution for X-ray pictures, achieved a sensitivity of over 98% and a specificity of over 95% for COVID-19 detection.

The COVID-Net architecture was proposed in [50]. The model was trained using an open resource called COVIDx, which included 13,975 X-ray pictures, despite the fact that only 266 of 358 patients were classified as COVID-19. The accuracy achieved was 93.3%.

In [51] author suggests Decompose, Transfer, and Compose (DeTraC), for the diagnosis of COVID-19 from chest X-ray images. The experiment employs an image dataset acquired from many hospitals across the globe. DeTraC can address any anomalies in the image dataset by exploring its class borders using a class decomposition method with an accuracy of 93.1% in the identification of COVID-19 X-ray images from healthy individuals and severe acute respiratory syndrome patients.

Another study [52] used a CNN based on capsule networks (CapsNet) for binary classification (COVID-19 vs. control) and multi-class classification (COVID-19 vs. pneumonia vs. control). Experiments were conducted on a dataset of 1050 X-ray pictures of COVID-19, 1050 pneumonia, and 1050 controls. The experiment obtained 97% accuracy for binary classification and 84% accuracy for multi-class classification using a tenfold cross-validation method.

The authors of [53] used transfer learning to develop a model for COVID-19 classification using the Xception network. Experiments were conducted on a database of 127 COVID-19, 500 controls, and 500 pneumonia cases collected from various sources, and achieved an accuracy of about 97%.

The authors in [54] present a technique called multi-kernel depthwise convolution (MD-Conv), which combines depthwise convolution kernels with varying filter widths into a single depthwise convolution layer. The tests were conducted on the chest X-ray dataset of 7470 individuals. MD-Conv had an AUC of 98.3% and an accuracy of 93.4% in diagnosing juvenile pneumonia.

In [55], a novel CNN model was proposed in this study with end-to-end training. A dataset containing 180 COVID-19 and 200 normal (healthy) chest X-ray images was used in the study’s experimentation. A classification accuracy of 91.6% was achieved as the performance measurement of the study.

An algorithm called BMO-CRNN using chest X-ray images was proposed in the paper [56] as a smart COVID-19 diagnostic model that uses a barnacle mating optimization (BMO) algorithm and a cascaded recurrent neural network (CRNN) model. This approach uses a set of CRNN hyperparameters, such as learning rate, batch size, activation function and epoch count to find the best possible values of these parameters. When it came time to run simulations, it showed that the BMO-CRNN model had shown the ideal performance of 97.01% sensitivity, an accuracy of 97.31%, and an F-value of 97.53%

The majority of the published research have utilized chest X-ray images to detect COVID-19, emphasizing the value of chest X-ray image analysis as a reliable tool for doctors and radiographers. Table 1 is an overview of the published research. However, it is known that neural network models require a large amount of data in order to be trained and tested. In the studies [35,36,37,39,41,42,43,44,45,46,47,49,51,52,53,55,56], researchers have worked with a small number of chest X-ray data (75–6200 data) for training and testing purposes of COVID-19 detection and achieved varying accuracy (89.2–98%). However, due to the lack of proper training of the models primarily because of using such limited datasets, the credibility of the outcome is questionable. Furthermore, in the studies [38,40,48,50,54], the models were trained on a large dataset (7470–79,500), which are considered reliable but did not provide accuracies in high nineties, being mostly between 91.5–95.7%. The studies also did not reflect on the compilation time of the implemented models, therefore the most efficient model could not be confidently identified either. Detecting COVID-19 in patients requires not only a highly accurate method but a fast and reliable one as well. To address this issue, a modified MobileNetV2 model is proposed that provides a higher accuracy rate in the shortest time compared to any existing CNN models. To achieve a large dataset for training and testing the model, 13,808 chest X-ray images of COVID-19 patients and healthy individuals were augmented to create a dataset of 52,000 images. To further ensure the credibility of the outcome and the model, a Wilcoxon signed-rank test to calculate *p*-value is performed.

## 3. Proposed Method

### 3.1. Dataset Preparation

**Figure 1:**
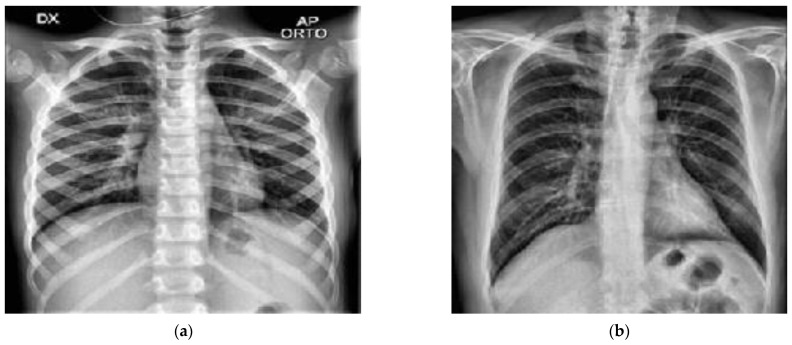
Chest X-ray image data samples. (**a**) Healthy; (**b**) COVID-19.

A chest X-ray database [57] was used to experiment with this study. This database is currently one of the popular public X-ray databases, containing 3616 COVID-19 cases along with 10,192 healthy, 6012 lung opacity and 1345 viral pneumonia images. However, only COVID-19 (3616) and healthy (10,192) X-ray images were extracted for this study. As a result, the dataset includes studies of COVID-19 and healthy individuals with a matrix resolution of 299 × 299 (two X-ray examples are shown in Figure 1). EnsNet [58], a system for scene-text removal, was used to remove annotations from certain images. EnsNet is capable of automatically removing all of the text or annotation from an image without any prior knowledge [59]. Data augmentation and image enhancement techniques are performed to enhance the quantity and variety of images given to the classifier for classification. Image augmentations used include horizontal flip, rotation, width shift and height shift on all the extracted data from the original dataset. As chest X-ray images are not vertically balanced, vertical flip was not applied. All augmentation parameters are shown in Table 2. After image augmentation, the dataset was increased to a larger dataset consisting of 26,000 COVID-19 and 26,000 healthy chest X-ray images. Besides, image enhancement applied Histogram equalization, Spectrum, Grays and Cyan. The N-CLAHE algorithm was then used to normalize pictures and highlight smaller features for machine learning classifiers to notice. Thereafter, the images were scaled down to the classifier’s standard resolution (for instance AlexNet was 256 × 256 pixels, whereas GoogLeNet was 224 × 224 pixels). After resizing the picture, the machine learning classifier used the enhanced (52,000) images in a ratio of 80% data for training, whereas 20% was used for testing. Table 3 shows the details of the dataset.

### 3.2. Model Selection

**Figure 2:**
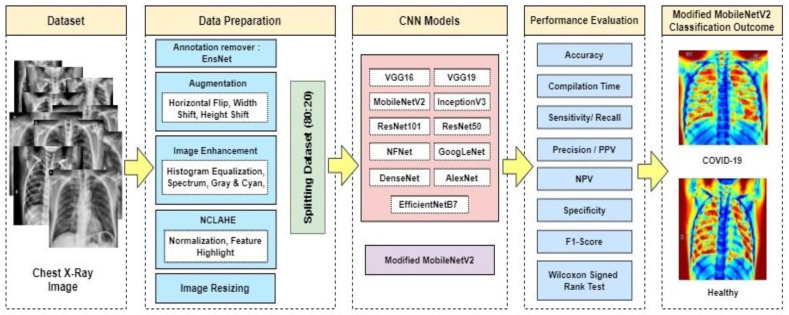
Proposed system diagram.

One of the main goals of this research is to obtain appropriate classification results utilizing freely available data (increased to high volume data by using enhancement techniques) with the combined transfer learning models. This research was undertaken to choose a CNN-based deep learning model that is appropriate for COVID-19 image classification investigation. The primary aim is to propose a modified novel deep-learning-based CNN model to gain the highest accuracy on a large volume of chest X-ray data with minimal compilation time and compare the modified novel approach (accuracy, efficiency, compilation time) with existing deep learning models on the same dataset. Figure 2 shows the system diagram of the experiment.

As a result, the focus was on models that are widely used, appropriate for transfer learning, and easily available in packaged forms via trustworthy public libraries such as Keras to identify the best suitable model for this study. Due to this, some basic models are compared with the proposed novel model. These are all available as Keras API models [60], and all of these enable transfer learning by pre-applying the ImageNet weights to the model.

1. VGG19 and VGG16

The Visual Geometry Group is abbreviated as VGG. VGG16 is built using multiple 33 kernel-sized filters sequentially (11 and 5 in the first and second convolutional layers, respectively). VGG’s input is set to a 224 × 244 RGB picture. The VGG-19 convolutional neural network was trained using over a million pictures from the ImageNet database. The network has a depth of 19 layers and is capable of classifying images of multiple classes. The VGG architectures’ primary concept is to keep the convolution size modest and constant while designing an extremely deep network.

2. InceptionV3

InceptionV3 makes use of label smoothing, factorized 7 × 7 convolutions, and an auxiliary classifier to transmit label information down the network, as well as batch normalization for sidehead layers. It features smaller convolutions for quicker training and lower grid size to overcome computational cost constraints. Numerous optimization methods have been proposed for an InceptionV3 model in order to relax the restrictions and facilitate model adaptability. Factorized convolutions, regularization, dimension reduction, and parallelized calculations are all included in the methods.

3. ResNet50 and 101

ResNet50’s architecture is divided into 4 stages. The network may accept an input image with a height, width of multiples of 32, and channel width. The network may accept an input image with a height, width of multiples of 32, and channel width Each ResNet architecture conducts initial convolution and max-pooling with a kernel size of 7 × 7 and 3 × 3, respectively. Each 2-layer block is replaced with this 3-layer bottleneck block in the 34-layer net, resulting in a 50-layer ResNet. A 101-layer ResNet is created by adding additional 3-layer blocks.

4. GoogLeNet

GoogLeNet is a deep convolutional neural network with 22 layers and almost 12× fewer parameters compared to Inception architecture. However, by adding more layers, the number of parameters grows, and the network may overfit. The pre-trained network accepts images with a resolution of 224 × 224. In GoogLeNet, global average pooling was utilized instead of a fully linked layer. The architecture makes use of the Activation, AveragePooling2D, and Dense layers.

5. MobileNetV2

MobileNetV2 introduces a new module with an inverted residual structure. With MobileNetV2, state-of-the-art object recognition and semantic segmentation are accomplished. MobileNetV2’s architecture begins with a fully convolutional layer with 32 filters and 19 residual bottleneck layers. Typically, the network requires 300 million multiply-add operations and utilizes 3.4 million parameters. Accuracy is increased by removing ReLU6 from the output of each bottleneck module.

6. AlexNet

AlexNet is made up of 5 convolutional layers, 3 max-pooling layers, 2 normalization layers, 2 fully connected layers, and 1 softmax layer. Each convolutional layer is composed of convolutional filters and a ReLU nonlinear activation function. Max pooling is accomplished using the pooling layers. Due to the existence of completely linked layers, the input size 224 × 224 × 3 is fixed. If the input picture is grayscale, it is converted to RGB by duplicating the single channel to create a three-channel RGB image. AlexNet’s total parameter count is 60 million, with a batch size of 128.

7. EfficientNet B7

To enhance performance, a new baseline network was created using the AutoML MNAS framework, which improves both accuracy and efficiency (FLOPS). The resultant architecture is comparable to MobileNetV2 and MnasNet in that it utilizes mobile inverted bottleneck convolution (MBConv), but is somewhat bigger owing to an increased FLOP budget. The basic network is then scaled up to create a family of models called EfficientNets. EfficientNetB7 does not include any pre-trained weights.

8. DenseNet 121

Each layer in a DenseNet design is directly linked to every other layer, resulting in the term Densely Connected Convolutional Network. There are L(L + 1)/2 direct connections between ‘L’ levels. The feature maps from previous layers are not averaged, but concatenated and utilized as inputs in each layer. As a result, DenseNets need fewer parameters than a comparable conventional CNN, which enables feature reuse by discarding duplicate feature maps. Dense Blocks, in which the size of the feature maps stays constant inside a block but the number of filters varies. These layers in between are referred to as Transition Layers and are responsible for downsampling the image by using batch normalization, 1 × 1 convolution, and 2 × 2 pooling layers.

9. NFNet

NFNets is an abbreviation for Normalizer-Free Networks. NFNets are a subclass of modified ResNets that achieve competitive accuracy in the absence of batch normalization. NFNets scales the activations at the start and end of the residual branch using two scalers (α and β). Scaled Weight Standardization is used in NFNets to prevent mean shift. Additionally, Adaptive Gradient Clipping was used to train NFNets with larger batch sizes and learning rates.

10. Modified MobileNetV2 (Novel Approach)

Modified MobileNetV2 is likewise a design suited for mobile as well as computer vision like MobileNetV2. To assist with computer vision, deep learning techniques are now being utilized in other areas including robotics, the Internet of Things (IoT), and Natural Language Processing. The modified MobileNetV2 model, as well as the CNN layers, are used to predict and categorize diseases in chest X-ray images in this study. The modified MobileNetV2 architecture includes a set of hidden layers based on a bottleneck residual block, as well as a depth-wise separable convolution that significantly lowers the number of parameters and results in a lightweight neural network that differs from typical convolution. The standard convolution is substituted with a depth-wise convolution with a single filter, followed by a depth-wise severable convolution with a pointwise convolution.

### 3.3. Modified MobileNet V2 Architecture

The modified MobileNetV2 that has been proposed is not only compact in size but also computationally efficient, leading to enhanced performance on both large and small data sets.

Seven convolutional layers formed the bulk of the bottleneck residual block in the modified model. The final two layers that were previously included in the initial generation of MobileNet: a depth-wise convolution filtering the inputs and a 1 × 1 pointwise convolution layer. Though, this layer 1 × 1 role has shifted. The main concept is to use 3 × 3 depth-separable convolution filters followed by 1 × 1 subsequent convolution filters instead of the usual 3 × 3. The new design uses fewer operations and parameters to achieve the same filtering and combining process as traditional convolution. In MobileNetV1, the pointwise convolution had to either double or maintain the number of channels. In MobileNetV2, pointwise convolution has the opposite effect: it decreases the number of available channels. The first new feature was introduced by the expansion layer. The expansion layer is a 1 × 1 convolution. Its function is to increase the number of channels in the image data before proceeding to depth-wise convolution. As a result, since it performs the reverse of the projection layer, this expansion layer always has more output channels than input channels. Table 4 shows the architecture of modified MobileNetV2.

The residual connection described in Algorithm 1 is a novel feature in the model building block. An expansion factor t is applied to the feature channels. For testing, modified MobileNetV2 with an input size of 224 × 224 was utilized. Modify the second convolution layer with kernel size (2, 2). At the first residual block, use stride 0.5 rather than 1. In the fifth residual block, use stride 2 rather than 1. RMSprop optimizer is added in the third convolution layer.
**Algorithm 1:** Proposed Modified MobileNetV2 Algorithm for COVID-19 Detection**Input:** 52,000 chest X-ray images. (80% train, 20% test data)**Output:** Result = COVID-19 positive or healthy**Step 1:** batch normalization, preprocessing, augmentation**Step 2:** Freeze the base layer and add proposed convolution layer with image size   224, kernel size (3, 3), optimizer = RMSprop, activation: ReLU**Step 3:** Feed the first residual convolution layer with kernel size (2, 2), activation =   ReLU, then average pooling, optimizer = RMSprop**Step 4:** Feed into the second residual convolution layer with kernel size (1, 1), stride = 2.   Average pooling, dropout, optimizer = none**Step 5:** Feed into the third residual convolution layer with kernel size (2, 2), stride = 1.   Max pooling, dropout, optimizer = RMSprop**Step 6:** Feed into the fourth and fifth residual convolution layer with kernel size (1, 1),   stride = 2. Max pooling, dropout, optimizer = none**Step 7:** Feed into sixth residual convolution layer with kernel size (2, 2), stride = 2, no   pooling, dropout, optimizer = RMSprop**Step 8:** Feed into seventh residual convolution layer with kernel size (2, 2), stride = 1,   average pooling, dropout, optimizer = RMSprop**Step 9:** Apply proposed layer with image size 224 × 224, kernel size (3, 3), optimizer =   RMSprop, activation: ReLu**Step 10:** Finding the accuracy, precision, f1 score, recall

The algorithm takes 52,000 chest X-ray images as its input and splits the dataset to 80% as training data and 20% as test data. The outcome obtained is the classification of the data as either COVID-19 positive or healthy.

At first, batch normalization, preprocessing and augmentation are applied to the dataset. The base layer is then frozen, and the proposed convolution layer with image size 224 × 224 pixel is added. Here, RMSprop optimizer and ReLU activation function are also used. The first residual convolution is fed with kernel size of (2, 2), ReLU activation function, average pooling and RMSprop optimizer. The second residual convolution layer is fed with kernel size of (1, 1), stride = 2, average pooling, dropout to handle overfitting but no optimizer. In the third residual convolution layer, kernel size of (2, 2), stride = 1, max pooling, dropout along with RMSprop optimizer is fed. Both the fourth and fifth residual convolution layer is fed with kernel size of (1, 1), stride = 2, max pooling, dropout to handle overfitting but no optimizer. No pooling function is fed into the sixth residual convolution layer but kernel size of (2, 2), stride = 2, dropout along with RMSprop optimizer was fed. The final residual convolution layer feeds on a kernel size of (2, 2), stride = 1, average pooling, dropout along with RMSprop optimizer. Next, the proposed layer is applied on images of 224 × 224 pixels with a kernel size of (3, 3), RMSprop optimizer and ReLU activation function. The final step of the algorithm is to find the accuracy, precision, f1 score, and recall of the outcome.

### 3.4. Model Implementation

A pre-trained network has been previously trained on a bigger dataset, which is usually adequate to develop a unique hierarchy from which features may be extracted. On tiny datasets, it performs better. Simoyan and Zisserman (2014) [61] created the VGG16 architecture, which is a prime example. The models used and described in the previous section are available as pre-packaged within Keras except for the novel approach. All models are firstly tuned using Keras-tune to determine the optimal hyperparameter ranges. The grid search approach is used, which is a popular method for parameter tuning. Initially picked the following at random:

Batch size = (50, 100, 150, 200, 250, 300)
Number of epochs = (100, 150, 200, 250, 300, 350, 400)
Learning rate = (0.0000001, 0.000001, 0.00001, 0.0001, 0.001, 0.01, 0.1)
Optimizer = (‘SGD’, ‘Adadelta’, ‘RMSprop’, ‘Adagrad’, ‘Adam’, ‘Nadam’, ‘Adamax’)

And below mentioned parameters are selected manually for all deep learning models

Hidden layer size = 8–96 neurons
Dropout = 0.1
Activation = (‘ReLU’ (hidden), ‘Sigmoid’ (final))
Kernel Size = 2 × 2

Furthermore, the value of parameters using the grid search method for all deep learning models was achieved. The overall data of parameters post tuning is shown in Table 5. Only in the novel method (modified MobileNetV2) applied all optimizers (‘SGD’, ‘Adadelta’, ‘RMSprop’, ‘Adagrad’, ‘Adam’, ‘Nadam’, ‘Adamax’) and picked the one (‘RMSprop’) that provides the highest accuracy.

For further analysis, every model was trained multiple times over a period of 150–400 epochs with precision, recall, f1 score, accuracy (training/testing) metrics, as well as training curves and confusion matrices, recorded.

The experiment was performed with learning rates ranging from 0.1 to 0.0000001, and the hidden layer size was changed from 8 to 96 neurons, as well as the learning rate. The batch size varied between 50 and 300. ImageNet weights were used to train each classifier. To avoid overfitting, the number of dropout sections was adjusted. For enhanced performance, all eleven models except the novel model epochs, optimizers, batch size and learning rate were chosen using grid search. Repeated training and testing were done with the chosen epochs and adjusted parameters to obtain performance scores. After that, the training and testing were performed with a different epoch and optimized hyperparameters in order to get performance scores.

According to the testing outcomes exhibited in Table 6, the novel method modified MobileNetV2 was more trainable on image classes (COVID-19, healthy) and provided more reliable highest results when compared to the other models.

### 3.5. Performance Evaluation Matrix

A confusion matrix was used to show the performance results [62]. The matrix is composed of the following elements:

True-positive (TP): refers to instances of COVID-19 that have been correctly classified.
False-positive (FP): refers to instances of COVID-19 that were erroneously classed as healthy.
True-negative (TN): refers to instances that are correctly classified as healthy.
False-negative (FN): refers to instances of COVID-19 that were erroneously categorized as healthy.

Using the elements of the matrix, model accuracy, precision, recall, F1 score, negative predicted values, and specificity are measured. In Equation (1), accuracy is the ratio of correctly identified COVID-19 and healthy cases from all samples.

(1) $$\mathrm{Accuracy}=\frac{\mathrm{TP}+\mathrm{TN}}{\mathrm{TP}+\mathrm{TN}+\mathrm{FP}+\mathrm{FN}}$$

In Equation (2), precision or positive predicted value (PPV) is the ratio of correctly identified COVID-19 cases from all samples classified as COVID-19.

(2) $$\mathrm{Precision}=\frac{\mathrm{TP}}{\mathrm{TP}+\mathrm{FP}}$$

In Equation (3), sensitivity or recall is the ratio of correctly identified COVID-19 cases from the sum correctly identified COVID-19 and incorrectly classified COVID-19 cases as healthy, i.e., all the original COVID-19 samples.

(3) $$\mathrm{Recall}=\frac{\mathrm{TP}}{\mathrm{TP}+\mathrm{FN}}$$

In Equation (4), F1 score is the overall measure of the accuracy of the model using precision and recall.

(4) $$F1-\mathrm{Score}=2\left(\frac{\mathrm{Precision}\times \mathrm{Recall}}{\mathrm{Precision}+\mathrm{Recall}}\right)$$

In Equation (5), negative predicted value (NPV) is the ratio of correctly identified healthy cases from all samples classified as healthy.

(5) $$\mathrm{NPV}=\frac{\mathrm{TN}}{\mathrm{TN}+\mathrm{FN}}$$

In Equation (6), specificity is the ratio of correctly identified healthy cases from the sum correctly identified healthy and incorrectly classified healthy cases as COVID-19, i.e., all the original healthy samples.

(6) $$\mathrm{Specificity}=\frac{\mathrm{TN}}{\mathrm{TN}+\mathrm{FP}}$$

Finally, to evaluate the validity and reliability of the findings, a statistical test of significance is done using a Wilcoxon signed–rank test (two-tailed). In this test, the *p*-value is determined to evaluate the statistical significance.

## 4. Results

### 4.1. Experiment Setup

All the experiments were performed on AMD Ryzen 7 3800 × 3.90 GHz 8 (32 MB L3 cache, memory speed = 3200 MHz) cores 16 threads processor with 64 GB of RAM and Anaconda 3 software environment. It is loaded with a GPU from the MD Radeon RX 580 series. Following many evaluations of eleven different models, the novel approach modified MobileNetV2 model was chosen as the best model with the highest accuracy. Modified MobileNetV2 took almost 5 days for training with this setup. The results were derived using Python and OpenCV. Scikit-learn and NumPy were used to test the model.

### 4.2. Experiment Outcome

**Figure 3:**
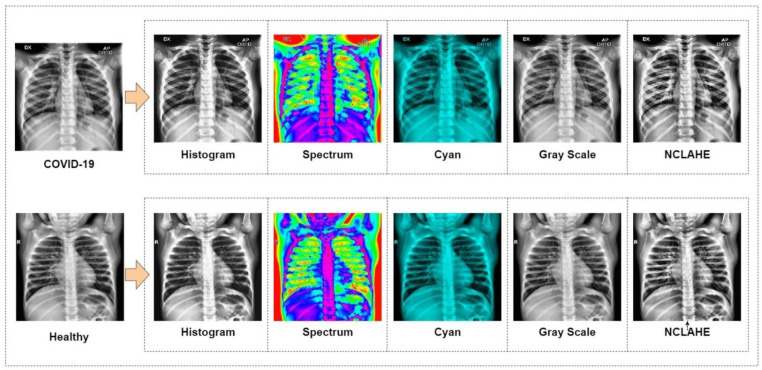
Prepared data for classification.

The suggested study used pre-trained CNN models and compared their performance to a new modified MobileNetV2 technique in order to get the highest performance for COVID-19 identification from 52,000 (26,000 COVID-19 and 26,000 healthy) chest X-ray images. Along with the proposed modified MobileNetV2, the VGG16, VGG19, MobileNetV2, ReseNet101, InceptionV3, ResNet50, GoogLeNet, AlexNet, EfficientNetB7, DenseNet and NFNet deep models were assessed in terms of performance and compilation time, since the purpose of the study is to find the most optimal model. Each model was fine-tuned and used epochs (100–400) and optimizers (suggest by grid search) to optimize the loss function with a learning rate of 0.000001, and the top 10 epochs were recorded and assessed. Before images were inputted into the neural network, they were downsampled to the optimal size for the models i.e., 256 × 256 for AlexNet. While 80% of the data was set aside for training, the remaining 20% was set aside for testing. In Figure 3, a sample of COVID-19 and healthy chest X-ray image data are presented. These refined data were fed into the CNN models for better accuracy of classification.

**Figure 4:**
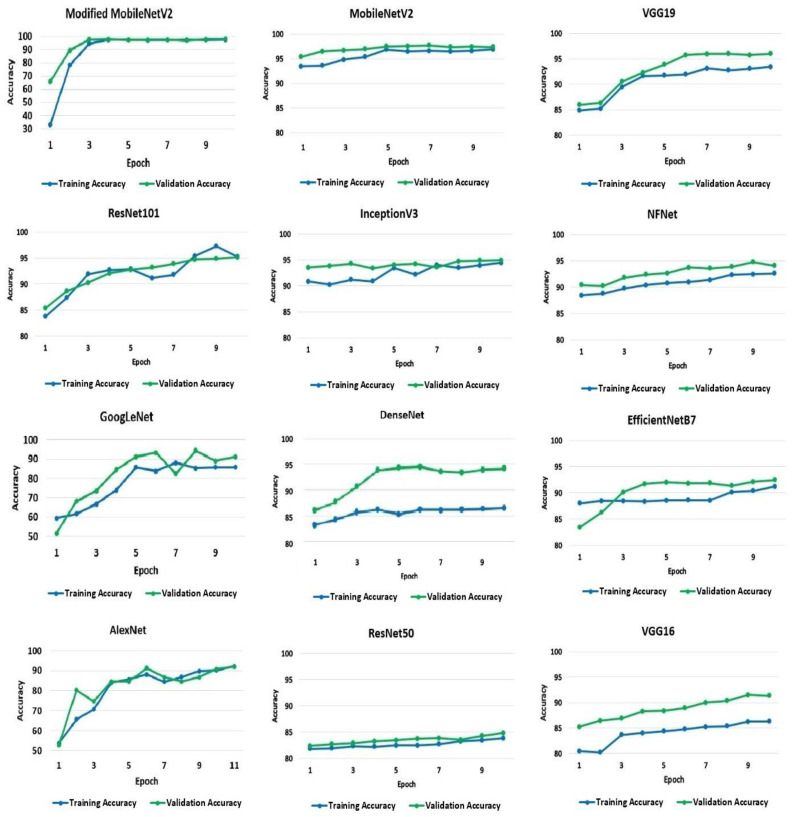
Training and validation accuracy of the models.

To begin with, the default validation set of 10% of the training set has been used. The accuracy and loss values acquired for the training and validation processes from the models were applied to the dataset. The classification training and validation accuracy scores were utilized to evaluate the performance of the models, and Figure 4 shows the resulting accuracy scores for the 10 recorded epochs. The modified MobileNetV2 proposed in the research achieved the highest accuracy for training and validation at 97% and 98%, respectively, followed by the pre-trained model, MobileNetV2. The MobileNetV2 model accuracy, when applied to the dataset, is 96% for the training set and 97% for the validation set. The VGG19 and ResNet101 models both achieved an identical test accuracy of 95%. Likewise, InceptionV3, NFNet, GoogLeNet, and DenseNet models all achieved a similar accuracy of 94%. The remaining models, EfficientNetB7, AlexNet, VGG16, and ResNet50, have a 92%, 91%, 91%, and 84% accuracy, respectively.

**Figure 5:**
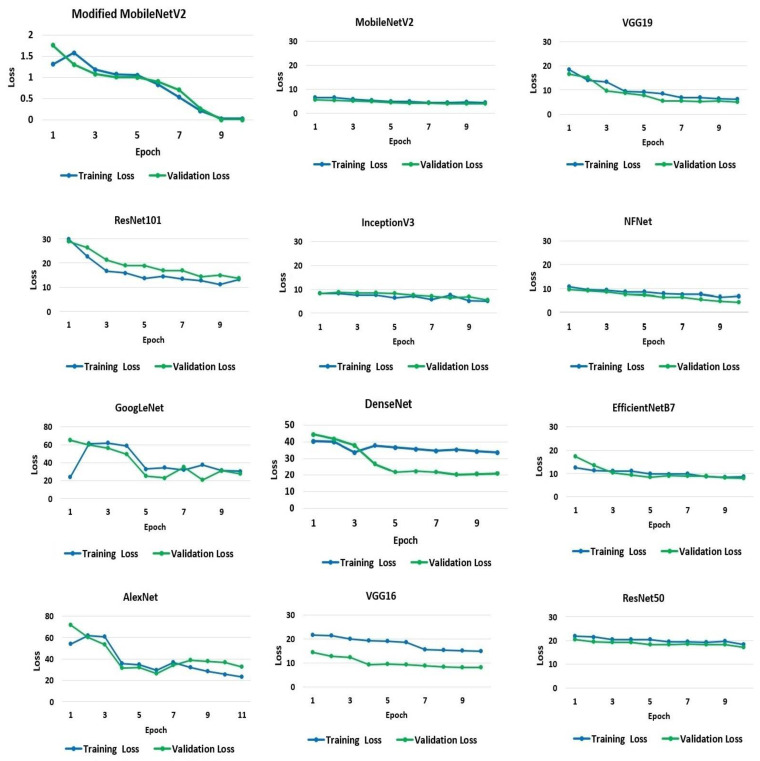
Loss in training and validation of the models.

A model’s loss value indicates how well or badly it performs after each iteration, depending on the model. Less loss means higher performance, unless a model has been over-fitted to its training data. The loss experienced by the models throughout the training and testing processes is shown and quantified in Figure 5. On average, the rate of loss decreased as the number of epochs increased. The average loss varied significantly across models. However, the suggested modified MobileNetV2 model results in the smallest loss of 0.0039%. MobileNetV2 has the second-lowest loss rate at 4% and 3% for the training and validation sets, respectively. The most significant loss is seen in GoogLeNet, Dense-Net, and AlexNet. The GoogLeNet has a loss rate of 23% and 20% for the training and validation datasets, respectively. AlexNet demonstrates losses of 23% and 31% from training and validation data, respectively. DenseNet exhibits a loss rate of 33% and 20% from the training and validation sets, respectively.

**Figure 6:**
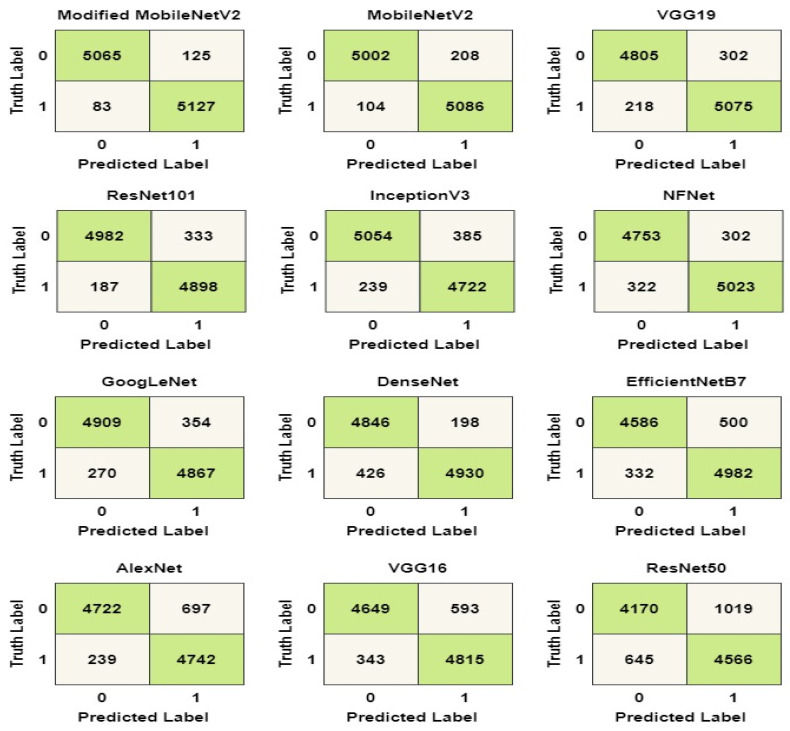
Confusion matrix of the models.

The confusion matrix for the models is shown in Figure 6 to aid in visualizing their overall performance. The test dataset contains 10,400 data samples, 5200 of which are COVID-19 samples and 5200 of which are healthy samples. COVID-19 and healthy instances are denoted by the labels ‘1’ and ‘0’, respectively. From the matrix, modified MobileNetV2 can successfully identify 5065 healthy samples and 5127 COVID-19 samples. In total, the model could accurately identify a total of 10,192 (98%) data samples from the test set. Similarly, MobileNetV2 models could accurately identify 5086 COVID-19 samples and 5002 healthy cases, amounting to 97% accuracy.

The confusion matrix in the Figure 6 was used to calculate the values of the labels TP, TN, FP, and FN. The accuracy, precision, recall, and F1 score of the models were determined using the values in Equations (1)–(6). The computed values for the performance metrics are shown in Table 7. As shown in the table, the modified MobileNetV2 has the best overall performance with a precision score of 97%, a recall score of 98%, a specificity score of 97%, NPV score of 98% and an F1 score of 97%.

Additionally, the compilation time is evaluated for the implemented models. Note that, high accuracy accompanied by low completion time makes a model more efficient. Table 8 shows the compilation time for each of the models for each epoch in seconds and a total compilation time in hours (h), minutes (min) and seconds (s). The final time was acquired by multiplying the time per epoch and epoch of each model. From the table, it is observed that the EfficientNetB7 model performs very poorly and has the highest completion time compared to the rest of the 11 models with 6 h, 45 min and 47 s. MobileNetv2 also has a comparatively higher compilation time than most of the models, even though the compilation time of each epoch is much lower. This is because the total epoch needed by the model to give the best prediction is comparatively high. Finally, it can be understood from the table that the proposed modified MobileNetV2 not only has the lowest compilation time with 2 h, 50 min and 21 s, but also the lowest total completion time even with the highest number of epochs (Table 5). That means the modified MobileNetV2 model gives the most optimal prediction result with the highest accuracy and lowest compilation time.

**Figure 7:**
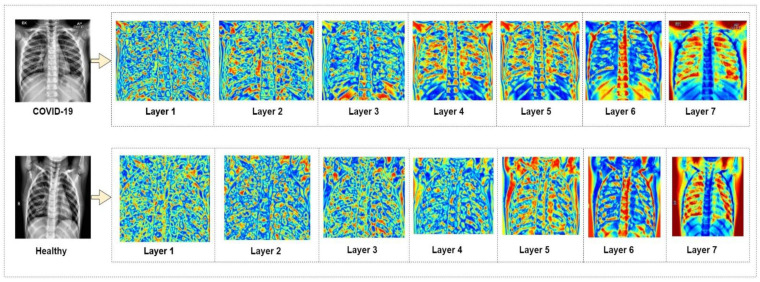
Heat map of modified MobileNetV2 classification.

It is observed from the performance measure comparison that the suggested modified MobileNetV2 model shows superior performance compared to the pre-trained CNN models. The model successfully can identify and tell apart COVID-19 and healthy chest X-ray images. In Figure 7, a heat map is presented that shows the classification process of the suggested model at each level of its architecture.

In Table 9, the performance measures of the modified MobileNetV2 are illustrated in detail. It shows the rate of performance scores in both the training and validation phase of the model. Overall, the training performance and testing performance show very high scores with 96% and 97% F1-scores.

A statistical test of significance was performed to validate the reliability of the findings to ascertain that the outcome did not occur by chance. To achieve so, the study followed the Wilcoxon signed-rank test and calculated *p*-value(s) among all models. The Wilcoxon signed-rank test is generally preferred as a non-parametric comparison between two samples [63]. This test is applied to compare two independent samples to perform a paired difference test of repeated measurements on a single sample. Subsequently, the result shows whether their population mean ranks differ or not. Table 10 depicts the results of the *p*-value for pair-wise comparison among the models. It is observed that modified MobileNet2 showed better performance than the rest of the models. To explain, the *p*-value between modified MobileNetV2 and MobileNetV2 is 0.01–0.001 (less than 0.05), which indicates that modified MobileNetV2 performed superiorly compared to MobileNet2, and the result is statistically significant.

## 5. Conclusions

The chest X-ray images (COVID-19, healthy) were mostly applied to analyze lung problems. The study attempts to understand the specific strengths and weaknesses of common deep learning models in order to identify COVID-19 with acceptable accuracy. This is critical for a doctor’s decision-making, since each has benefits and drawbacks. Furthermore, when time, resources, and the patient’s condition are restricted, the doctor may be forced to make a choice based on only one modality. In this work, deep learning techniques were used for automatic COVID-19 detection from chest X-ray images. For this, twelve different models were implemented. Among them, eleven models are existing CNN models while the last one is modified MobileNetV2, a novel approach suggested in the study for more accurate classification with the least compilation time. In this study, authors have shown that the existing methods, when trained and tested on a large dataset, are outperformance by the proposed modified model in COVID-19 detection. The classification accuracy of the modified MobileNetV2 model is 98% in 2 h 50 min 21 s. The precision, recall, sensitivity, and F1 score of the model are 97%, 98%, 97% and 97%, respectively. Compared to the proposed model, the highest performance achieved by the existing models is from MobileNetV2. The existing MobileNetV2 model has an accuracy of 97% in classifying COVID-19 and healthy chest X-rays in 5 h 42 min 34 s. The precision, recall, sensitivity, and F1 score of this model are 96%, 97%, 96% and 96%, respectively. Furthermore, the Wilcoxon signed-rank test done in the study confirms the validity of the findings. These findings will assist doctors in choosing suitable models for various image analysis methods, which will be important when time and resources are limited in a pandemic scenario like the present COVID-19. As future work, the proposed method could be implemented on a dataset with more classes of pulmonary diseases such as asthma, chronic obstructive pulmonary disease, pulmonary fibrosis, pneumonia, lung cancer and COVID-19. Additionally, from the literature review, it was observed that there is a lack of proper feature extraction processes from image data. Therefore, a feature extraction technique will also be included in future work.

## Figures and Tables

**Table 1:** Overview of studies using deep learning approaches with their performance for COVID-19 case detection.

Reference	Number of Image Samples	Classes	Architectures	Best Performing Architecture	Performance/Accuracy
[35]	1428	3	VGG19, MobileNetV2, Inception, Xception, InceptionResNetV2	MobileNetV2	Acc = 96.78%
[36]	204	2	VGG16 + Resnet50	VGG16 + Resnet50 + custom CNN	Acc = 89.2%
[37]	100	2	ResNet50, InceptionV3 and InceptionRes-NetV2	ResNet50	Acc = 98%
[38]	21,152	2	CNN	CNN	Acc = 94.64%
[39]	5184	2	ResNet18, ResNet50, SqueezeNet, DenseNet-121	SqueezeNet	Sensitivity = 98%, Specificity = 92.9%
[40]	13,975	2	COVID-CAPS	COVID-CAPS	Acc = 95.7%,
[41]	400	2	VGG16, InceptionResNetV2, ResNet50, DenseNet201, VGG19, MobilenetV2, NasNetMobile, and ResNet15V2	NasNetMobile	Acc = 93.94%
[42]	75	2	VGG19, Xception, ResNetV2, DenseNet201, InceptionV3, MobileNetV2, InceptionResNetV2	VGG19, DenseNet	F1 scores = 0.91
[43]	1127	2	Modified Darknet	Modified Darknet	Acc = 98%
[44]	1257	3	Xception	Xception	Acc = 94%
[45]	2356	3	ACoS system	ACoS	Acc = 91.33%
[46]	6100	3	SVM, LR, nB, DT, and kNN + VGG16, ResNet50, MobileNetV2, DenseNet121	Mean result	Acc = 98.5%
[47]	1428	2	VGG16	VGG16	Acc = 96%
[48]	79,500	3	Grad-CAM	Grad-CAM	Acc = 91.5%
[49]	6200	4	CSEN-based classifier	CSEN-based Classifier	Sensitivity = 98% Specificity = 95%
[50]	13,975	19	COVID-Net	COVID-Net	Acc = 93.3%
[51]	196	3	DeTrac	Detrac	Acc = 93.1%
[52]	3150	3	CapsNet	CapsNet	Acc = 97%
[53]	1127	3	Xception	Xception	Acc = 97%
[54]	7470	2	MD-Conv	MD-Conv	Acc = 93.4%
[55]	380	2	Novel CNN Model	Novel CNN Model	Acc = 91.6%
[56]	247	2	BMO-CRNN	BMO-CRNN	Sensivity = 97.01% Acc = 97.31% F-value = 97.53%

**Table 2:** Parameters of data augmentation.

Augmentation Technique	Range
Horizontal flip	True
Rotation range	10
Width shift range	0.1
Height shift range	0.1
Vertical flip	False

**Table 3:** Dataset Description.

Features	Values
Total Number of Images	52,000
Disease Types	2
Dimension (Size in Pixel)	Classifier’s Resolution (i.e., AlexNet is 256 × 256 pixels)
Color Grading	Grays, Cyan, Spectrum
COVID-19 Images	26,000 (After Augmentation)
Healthy Images	26,000 (After Augmentation)
Training Images	41,600
Testing Images	10,400

**Table 4:** Layers of modified MobileNetV2 Architecture.

Input	Operator	t	c	*n*	s
224 × 224 × 3	Con 2d	1	32	1	2
224 × 224 × 3 Average pool (3, 3)	bottleneck	3	16	1	1
128 × 128 × 3 Average pool (3, 3)	bottleneck	3	24	1	2
128 × 128 × 3 Maxpool (2, 2)	bottleneck	3	32	2	2
128 × 128 × 3 Maxpool (1, 1)	bottleneck	3	64	2	1
64 × 64 × 3 Maxpool (1, 1)	bottleneck	3	96	1	2
64 × 64 × 3	bottleneck	3	160	1	1
64 × 64 × 3	bottleneck	3	320	1	2
56 × 56 × 3	Con 2d	-	k	-	-

**Table 5:** Model final parameters.

Model	Batch Size	Number of Epochs	Hidden Layer Size	Dropout	Learning Rate	Activation	Optimizer	Kernel Size
VGG16	150	200	8–96 neurons	0.1	0.00001	ReLu sigmoid	Adam	2 × 2
VGG19	150	200	8–96 neurons	0.1	0. 0001	ReLu sigmoid	RMSprop	2 × 2
InceptionV3	200	300	8–96 neurons	0.1	0.0001	ReLu sigmoid	Nadam	2 × 2
ResNet50	100	200	8–96 neurons	0.1	0.001	ReLu sigmoid	Adamax	2 × 2
ResNet101	250	300	8–96 neurons	0.1	0.0001	ReLu sigmoid	Adam	2 × 2
GoogLeNet	50	150	8–96 neurons	0.1	0.0001	ReLu sigmoid	SGD	2 × 2
MobileNetV2	250	300	8–96 neurons	0.1	0.01	ReLu sigmoid	RMSprop	2 × 2
AlexNet	100	150	8–96 neurons	0.1	0.00001	ReLu sigmoid	Adadelta	2 × 2
EfficientNet B7	200	300	8–96 neurons	0.1	0.000001	ReLu sigmoid	Adamax	2 × 2
DenseNet121	200	350	8–96 neurons	0.1	0.00001	ReLu sigmoid	Adagrad	2 × 2
NFNet	150	250	8–96 neurons	0.1	0.0001	ReLu sigmoid	Adadelta	2 × 2
Modified MobileNetV2 (Proposed Method)	300	400	8–96 neurons	0.1	0.0000001	ReLu	RMSprop	2 × 2

**Table 6:** Model performance summary.

Model	Image Mode & Training Records	Testing F1 Score
VGG16	X-ray No overfitting evident (dropout Apply)	0.87
VGG19	X-ray No overfitting evident (dropout Apply)	0.88
InceptionV3	X-ray No overfitting evident (dropout Apply)	0.91
ResNet50	X-ray No overfitting evident (dropout Apply)	0.82
ResNet101	X-ray No overfitting evident (dropout Apply)	0.93
GoogLeNet	X-ray No overfitting evident (dropout Apply)	0.90
MobileNetV2	X-ray No overfitting evident (dropout Apply)	0.95
AlexNet	X-ray No overfitting evident (dropout Apply)	0.87
EfficientNetB7	X-ray No overfitting evident (dropout Apply)	0.92
DenseNet121	X-ray No overfitting evident (dropout Apply)	0.94
NFNet	X-ray No overfitting evident (dropout Apply)	0.92
Modified MobileNetV2 (Proposed Method)	X-ray No overfitting evident (dropout Apply)	0.98

**Table 7:** Model performance summary.

Model	Sensitivity/Recall	Precision/PPV	Accuracy	NPV	Specificity	F1 Score
Modified MobileNetV2	98%	97%	98%	98%	97%	97%
MobileNetV2	97%	96%	97%	97%	96%	96%
VGG19	95%	94%	95%	95%	93%	94%
ResNet101	96%	93%	95%	96%	93%	94%
InceptionV3	95%	92%	94%	95%	92%	93%
NFNET	94%	94%	94%	93%	93%	94%
GoogLeNet	94%	93%	94%	94%	93%	93%
DenseNet121	92%	96%	94%	91%	96%	93%
EfficientNetB7	93%	90%	92%	93%	90%	91%
AlexNet	95%	87%	91%	95%	97%	90%
VGG16	93%	89%	91%	93%	98%	90%
ResNet50	87%	81%	84%	86%	80%	83%

**Table 8:** Compilation time of the models.

Model	Compilation Time
VGG19	5 h 18 min 14 s
VGG16	6 h 27 min 7 s
MobileNetV2	5 h 42 min 34 s
InceptionV3	7 h 6 min 41 s
ResNet50	6 h 1 min 22 s
AlexNet	4 h 54 min 3 s
google	4 h 57 min 52 s
ResNet101	6 h 18 min 18 s
EfficientNetB7	6 h 45 min 47 s
DenseNet	6 h 42 min 35 s
NFNET	6 h 24 min 26 s
Modified mobilenetv2	2 h 50 min 21 s

**Table 9:** Modified MobileNetV2 model performance on training and validation set.

Modified MobileNetV2	Sensitivity/ Recall	Precision/ PPV	Accuracy	NPV	Specificity	F1 Score
Train	97%	96%	97%	97%	96%	96%
Validation	98%	97%	98%	98%	97%	97%

**Table 10:** Wilcoxon signed-rank test (two-tailed).

No.	Pairwise Model Comparison	*p* Value	Significance < 0.05
1	Modified MobileNetV2 versus VGG16	0.0001	Yes
2	Modified MobileNetV2 versus VGG19	0.0117	Yes
3	Modified MobileNetV2 versus InceptionV3	0.0001	Yes
4	Modified MobileNetV2 versus ResNet50	0.0001	Yes
5	Modified MobileNetV2 versus ResNet101	0.0030	Yes
6	Modified MobileNetV2 versus GoogLeNet	0.0001	Yes
7	Modified MobileNetV2 versus MobileNetV2	0.0028	Yes
8	Modified MobileNetV2 versus AlexNet	0.0001	Yes
9	Modified MobileNetV2 versus EfficientNetB7	0.0001	Yes
10	Modified MobileNetV2 versus DenseNet121	0.0068	Yes
11	Modified MobileNetV2 versus NFNet	0.0023	Yes

## Data Availability

https://www.kaggle.com/tawsifurrahman/covid19-radiography-database?fbclid=IwAR0rw_prTvf9R0zInrJQkTFazeBaESxh3rB6otdrPdAWJDonEbIl2Nf6epk (accessed on 10 September 2021).
